# Clinical characteristics and outcomes of Stanford type B aortic intramural hematoma: A single centre experience

**DOI:** 10.3389/fsurg.2022.1071600

**Published:** 2023-01-05

**Authors:** Zhigong Zhang, Feng Lin, Zhipeng He, Haoran Wang, Xingyang Zhu, Tingting Cheng

**Affiliations:** ^1^ Department of General Surgery, The First Affiliated Hospital of Anhui Medical University, Hefei, China; ^2^ Anhui Public Health Clinical Center, Hefei, China; ^3^ Department of General Surgery, The Second Affiliated Hospital of Nanchang University, Nanchang, China

**Keywords:** intramural hematoma, TEVAR, medical management, aortic dissection, maximum aortic diameter

## Abstract

**Objective:**

To compare the clinical characteristics of Stanford type B aortic intramural hematoma (IMH) and Stanford type B aortic dissection (AD), and to identify the differences between thoracic endovascular aortic repair (TEVAR) and medical management (MM) in the Stanford type B IMH patients.

**Methods:**

A retrospective observational study was conducted in patients treated between January 2015 and December 2016. The clinical characteristics and CT images of patients with type B IMH and type B AD were compared, and the clinical characteristics and CT images of patients in the type B IMH group who were treated with TEVAR and MM were compared.

**Results:**

A total of 176 patients were included in this study, including 62 patients of type B IMH and 114 patients of type B AD. Five patients died in the IMH group and three in the AD group. The proximal hematoma or entry tear in both groups was mainly located in the descending aorta, and the proportion of the iliac artery involved in the AD group was significantly higher than that in the IMH group (31.6% vs. 8.1% *P* < 0.05). There were 50 MM patients and 12 TEVAR patients in the IMH group. No death occurred in the TEVAR group, while five patients in the MM group died. Seven patients in the MM group had disease progression vs. 12 in the TEVAR group (*P* < 0.05). The patients in the TEVAR group had more intima lesions than those in the MM group (83.3% vs. 30.0%, *P* < 0.05). TEVAR group involved more iliac artery hematoma than MM group (33.3% vs. 2.0%, *P* < 0.05). The maximum thickness of hematoma in TEVAR group was 14.9 ± 3.4 mm, which was significantly larger than that of MM group (10.2 ± 2.8 mm) (*P* < 0.05).

**Conclusion:**

In the diagnosis of IMH, patients' symptoms and high-risk signs of CTA should be paid attention to. TEVAR therapy should be actively considered on the basis of effective medical management when there are intima lesions (ULP/PAU), increased aortic diameter and hematoma thickness, extensive hematoma involvement, and pleural effusion.

## Introduction

Aortic intramural hematoma (IMH) has similar clinical manifestations to aortic dissection (AD) and penetrating aortic ulcer (PAU), so it is called acute aortic syndrome (AAS), of which IMH accounts for about 10%–30% of the total (1). In 1920, Krukenberg et al. first described IMH as “aortic dissection without intimal rupture” (2). Similar to AD, IMH can be divided into Stanford type A (hematoma involving the ascending and/or descending aorta) and Stanford type B (hematoma involving only the descending aorta), with the latter accounting for 50%–85% (3).

The initiating factors leading to the formation of IMH have not yet been fully clarified, but hypertension, atherosclerosis and smoking undoubtedly play an important role (4). The current mainstream view is that the hemorrhage in the aortic wall originates from the spontaneous rupture of the aortic vasa vasorum, resulting in the formation of a hematoma. Some scholars also believe that the tiny breach on the aortic intima or PAU is an important reason for the formation of IMH. Other possible mechanisms include pathological proliferation and spontaneous rupture of microvessels in atherosclerotic plaques (5). After an intermural hemorrhage, the aortic wall becomes weak, but the intima of the artery remains intact without rupture or internal diaphragm formation.

The clinical manifestations of IMH patients are similar to AD, mainly with the sudden onset of chest and back laceration pain, and most patients have a history of hypertension. CTA is the preferred method for imaging examination, which can not only confirm the diagnosis, but also know the hematoma range, diameter and whether there are microscopic lesions in the intima, providing a basis for subsequent treatment (6). It is generally believed that for Stanford type A IMH, surgery should be performed as soon as possible to prevent severe complications. For Stanford type B IMH, most patients can choose medical management (MM). When the IMH disease progresses, surgical intervention should be considered, and thoracic endovascular aortic repair (TEVAR) is the preferred treatment method (7).

Relevant studies indicate that 88% of type A IMH patients will progress to AD, while the proportion of type B IMH patients will progress to AD is 3%–14%. The natural course of IMH is mostly stable or absorbed after conservative treatment, but 15%–20% of patients will progress to AD or even aortic rupture, and other complications include pleural effusion and cardiac tamponade (1). This study will compare the clinical characteristics of type B IMH and type B AD, as well as the disease progression and outcomes of patients with type B IMH in the TEVAR group and MM group, in order to analyze the characteristics of the disease, CT image characteristics, treatment methods and prognosis.

## Materials and methods

### Patients and study design

In this study, patients admitted to the Department of Vascular Surgery of the First Affiliated Hospital of Anhui Medical University from January 2015 to December 2016 were collected, including 114 patients with type B AD and 62 patients with type B IMH. Complicated or high-risk type B AD were treated with TEVAR. All type B IMH patients were initially treated with medication on admission, primarily to control blood pressure, heart rate and pain. TEVAR treatment was actively considered for complicated cases of type B IMH in our center. PAU, ulcer like projection (ULP), intramural blood pooling were subjects for elective TEVAR. 68 patients in the AD group were treated with TEVAR, 12 patients in the IMH group were treated with TEVAR and 50 patients were treated with MM. This study was approved by the ethics committee of our hospital, and all patients gave informed consent. The exclusion criteria of this study included: transfer, withdrawal from treatment, severe complications and short survival period. A total of five patients in the type B AD group were excluded, and three patients in the type B IMH group were excluded.

### Data collection and analysis

This study firstly compared the clinical characteristics and CT image differences between type B IMH and type B AD patients, and then compared the clinical and CT image characteristics of MM group and TEVAR group in type B IMH patients.The collected indicators included demographic data, past history, chronicity classification, onset symptoms, positive findings in physical examination, CT imaging characteristics, treatment method, therapeutic outcome, in-hospital complications, in-hospital mortality, etc.

### Definitions and follow-up

All patients were evaluated with CTA on admission. The diagnostic criteria for AD is a typical double-channel aorta with a visible intimal tear or flap. Typical IMH on CTA imaging showed smooth, crescent-shaped aortic artery wall (CT value: 60–80 HU) or thickened annular aortic wall (>5 mm) with longitudinal extension, without intimal laceration or double-lumen flow concave (4). CTA was followed up once before discharge, three months after discharge, 12 months after discharge and 24 months after discharge. High-risk type B AD refers to the presence of any of the following risk factors: refractory pain, refractory hypertension, bloody pleural effusion, aortic diameter >40 mm, false lumen diameter >22 mm, radiographic only malperfusion, entry tear: lesser curve location, and readmission. Complicated type B AD include rupture and malperfusion. Complicated type B IMH was defined as recurrent pain, enlarged or thickened hematoma, mal-perfusion, maximum aortic diameter ≥55 mm, evolution to classic dissection or aneurysm, aortic rupture or impending rupture.

### Statistical analysis

Statistical analysis was performed with SPSS, release 19.0 for Windows. Categorical variables were compared using χ^2^ tests or Fisher's exact test. Continuous variables were compared using Student's *t* test or Mann-Whitney U-test. All reported *P* values are two-sided, and *P* values <0.05 were considered to be statistically significant.

## Results

### Comparison of clinical characteristics of type B IMH and type B AD

A total of 176 patients were included in this study, including 62 cases of type B IMH and 114 cases of type B AD. In the type B IMH group, there were 40 males and 22 females. In the type B AD group, there were 96 males and 18 females. There was a significant difference in gender composition between the two groups, and the proportion of males in the type B AD group was significantly higher (84.2% vs. 64.5%; *P* = 0.003). The average age of the type B IMH group was 68.4 ± 12.3 years, which was significantly higher than that of the type B AD group (58.3 ± 11.6 years) (*P* = 0.031). The risk factors included hypertension, diabetes mellitus, dyslipidemia, smoking history, coronary heart disease, pulmonary disease and chronic renal insufficiency. The proportion of chronic renal insufficiency in type B AD group was significantly higher than that in type B IMH group (15.8% vs. 3.2%; *P* = 0.012). Chronicity classification was formulated by Society for Vascular Surgery/Society of Thoracic Surgeons (SVS/STS), including hyperacute (<24 h); acute (1 to 14 days); subacute (15 to 90 days); and chronic (>90 days). The difference in chronicity classification between the two groups was not statistically significant. The initial symptoms of both groups were mainly chest and back pain, and other symptoms included abdominal pain, loss of consciousness, dyspnea, etc. The comparison of malperfusion in type B AD and type B IMH patients included lower limb, mesenteric, celiac and renal malperfusion. The renal malperfusion in type B AD group was significantly higher than that in type B IMH group (17.5% vs. 4.8%; *P* = 0.017). Serum creatinine in the type B AD group was also higher than that in the IMH group, but the difference between the two groups was not statistically significant (89.1 ± 31.1 vs. 82.8 ± 30.9; *P* = 0.200). In terms of admission hemodynamics, there was no significant difference in systolic blood pressure, diastolic blood pressure and heart rate between the two groups. 12 patients in the type B IMH group were finally treated with TEVAR, and 68 patients in the type B AD group were treated with TEVAR. There was no significant difference in in-hospital complications between the two groups, mainly including pulmonary infection, myocardial infarction, stroke, cardiac tamponade, and aortic rupture. Five patients died in the type B IMH group (three died of aortic rupture, one died of cerebral infarction, and one died of esophageal cancer), and three patients died in the type B AD group (two postoperatively from aortic rupture and one intraoperatively). In-hospital mortality in the type B IMH group was higher than in the type B AD group, but there was no significant difference between the two groups (8.1% vs. 2.6%; *P* = 0.098). All these outcomes are depicted in Table 1.

**Table 1:** Clinical characteristics of type B IMH and type B AD.

Category	Type B IMH	Type B AD	*P* value
Number of patients	62	114	
Male gender	40 (64.5)	96 (84.2)	0.003
Age, years	68.4 ± 12.3	58.3 ± 11.6	0.031
Risk factors			
Hypertension	58 (93.5)	106 (93.0)	1.000
Diabetes mellitus	4 (6.5)	12 (10.5)	0.369
Dyslipidemia	14 (22.6)	32 (28.1)	0.429
History of smoking	23 (37.1)	59 (51.8)	0.063
Coronary heart disease	5 (8.1)	15 (13.2)	0.309
Pulmonary disease	3 (4.8)	9 (7.9)	0.649
Chronic renal insufficiency, eGFR < 60 ml/min/1.73 m^2^	2 (3.2)	18 (15.8)	0.012
Chronicity classification^a^			
Hyperacute	25 (40.3)	33 (28.9)	0.125
Acute	19 (30.6)	30 (26.3)	0.540
Subacute	18 (29.0)	50 (43.9)	0.054
Chronic	0 (0)	1 (0.9)	1.000
Initial symptom			
Chest and back pain	45 (72.6)	90 (78.9)	0.340
Abdominal pain	14 (22.6)	18 (15.8)	0.265
Loss of consciousness	1 (1.6)	3 (2.6)	1.000
Dyspnea	1 (1.6)	2 (1.8)	1.000
Other	1 (1.6)	1 (0.9)	1.000
Malperfusion			
Lower limb	1 (1.6)	6 (5.3)	0.435
Mesenteric	1 (1.6)	4 (3.5)	0.804
Celiac	0 (0)	2 (1.8)	0.541
Renal	3 (4.8)	20 (17.5)	0.017
Serum creatinine, umol/l	82.8 ± 30.9	89.1 ± 31.1	0.200
Hemodynamics on admission			
SBP, mm Hg	142.0 ± 35.1	150.1 ± 37.5	0.064
DBP, mm Hg	92.4 ± 19.5	95.5 ± 18.4	0.231
Heart rate	90.7 ± 16.8	91.8 ± 21.5	0.346
TEVAR	12 (19.3)	68 (59.6)	<0.001
In-hospital complications			
Pulmonary infection	3 (4.8)	5 (4.3)	1.000
MI	1 (1.6)	3 (2.6)	1.000
Stroke	1 (1.6)	2 (1.8)	1.000
Cardiac tamponade	1 (1.6)	1 (0.9)	1.000
Aortic rupture	3 (4.8)	2 (1.8)	0.483
In-hospital mortality	5 (8.1)	3 (2.6)	0.098

AD, aortic dissection; DBP, diastolic blood pressure; IMH, intramural hematoma; MI, myocardial infarction; SBP, systolic blood pressure; TEVAR, thoracic endovascular aortic repair.

^a^ Hyperacute, <24 h; acute, 1 to 14 days; subacute, 15 to 90 days; and chronic, >90 days. Society for Vascular Surgery/Society of Thoracic Surgeons (SVS/STS) chronicity classification of aortic dissection.

Categorical variables are presented as number (%). Continuous variables are presented as mean ± standard deviation.

### Comparison of CT imaging of type B IMH and type B AD

**Figure 1:**
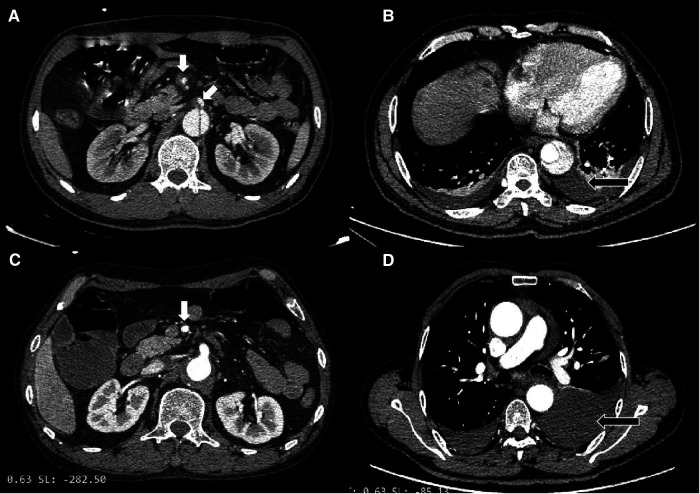
(**A**) Type B AD with superior mesenteric artery malperfusion. (**B**) Type B AD with pleural effusion. (**C**) Type B IMH with superior mesenteric artery malperfusion. (**D**) Type B IMH with pleural effusion.

The CT imaging differences between type B IMH and type B AD groups were further compared. From the initial CTA at the time of admission, the proximal hematoma or rupture in both groups was mainly located in the descending aorta. In terms of the extent of disease involvement, the proportion of type B AD involving the iliac artery was significantly higher than that in the type B IMH group (31.6% vs. 8.1%; *P* < 0.001). There was no significant difference in the maximum diameter of the aorta between the two groups, and the maximum thickness of the intramural hematoma in the type B IMH group was 12.7 ± 5.3 mm on average. There were 15 and 25 patients with pleural effusion in the type B IMH group and type B AD group, respectively (24.2% vs. 21.9; *P* = 0.732). All these outcomes are depicted in Table 2. Imaging data of malperfusion and pleural effusion in type B AD and type B IMH patients can be seen in Figure 1.

**Table 2:** Ct imaging of type B IMH and type B AD.

Category	IMH	AD	*P* value
Number of patients	62	114	
Location of proximal hematoma or entry tears			
Descending aorta	58 (93.5)	110 (96.5)	0.605
Abdominal aorta	4 (6.5)	4 (3.5)	0.605
Distal location of the lesion			
Descending aorta	16 (25.8)	14 (12.3)	0.023
Abdominal aorta	41 (66.1)	64 (56.1)	0.197
Iliac artery	5 (8.1)	36 (31.6)	<0.001
MAD, mm	41.2 ± 7.5	39.4 ± 6.9	0.146
Maximum haematoma thickness, mm	12.7 ± 5.3	/	/
Pleural effusion	15 (24.2)	25 (21.9)	0.732

AD, aortic dissection; IMH, intramural hematoma; MAD, maximum aortic diameter.

Categorical variables are presented as number (%). Continuous variables are presented as mean ± standard deviation.

### Comparison of clinical characteristics between TEVAR group and MM group in type B IMH

Patients with type B IMH were divided into TEVAR group and MM group, and the clinical characteristics of the two groups were compared. There was no significant difference in gender composition and age between the two groups. Among the risk factors, the proportion of patients with dyslipidemia in the TEVAR group was significantly higher (66.7% vs. 12.0%; *P* < 0.001). The difference in chronicity classification between the two groups was not statistically significant between hyperacute phase and acute phase. A higher proportion of patients in the MM group were in the subacute phase compared with the TEVAR group (36.0% vs. 0%; *P* = 0.035). The initial symptoms were mainly chest and back pain, and one patient in the TEVAR group had loss of consciousness. There were no significant differences in systolic blood pressure, diastolic blood pressure, and heart rate between the two groups at admission. In terms of blood pressure fluctuation, the maximum daily increase of blood pressure in the TEVAR group was significantly higher than that in the MM group (32.1 ± 7.8 vs. 20.4 ± 9.4; *P* = 0.045). There was no significant difference in in-hospital complications between the two groups, mainly including pulmonary infection, myocardial infarction, stroke, cardiac tamponade, and aortic rupture. There were no deaths during hospitalization in the TEVAR group, compared with five deaths in the MM group (The reason is mentioned above). One-year mortality was higher in the MM group than in the TEVAR group, but the difference was not significant (12.0% vs. 8.3%; *P* = 1.000). All these outcomes are depicted in Table 3.

**Table 3:** Clinical characteristics of TEVAR group and MM group.

Category	MM	TEVAR	*P* value
Number of patients	50	12	
Male gender	33 (66.0)	7 (58.3)	0.513
Age, years	63.7 ± 12.7	61.0 ± 15.1	0.527
Risk factors			
Hypertension	48 (96.0)	10 (83.3)	0.166
Diabetes mellitus	2 (4.0)	2 (16.7)	0.166
Dyslipidemia	6 (12.0)	8 (66.7)	<0.001
History of smoking	17 (34.0)	6 (50.0)	0.485
Coronary heart disease	3 (6.0)	2 (16.7)	0.246
Pulmonary disease	2 (4.0)	1 (8.3)	0.482
Renal insufficiency, eGFR < 60 ml/min/1.73 m^2^	1 (2.0)	1 (8.3)	0.352
Chronicity classification^a^			
Hyperacute	19 (38.0)	6 (50.0)	0.665
Acute	13 (26.0)	6 (50.0)	0.204
Subacute	18 (36.0)	0 (0)	0.035
Chronic	0 (0)	0 (0)	/
Initial symptom			
Chest and back pain	35 (70.0)	10 (83.3)	0.569
Abdominal pain	13 (26.0)	1 (8.3)	0.352
Loss of consciousness	0 (0)	1 (8.3)	0.194
Dyspnea	1 (2.0)	0 (0)	1.000
Other	1 (2.0)	0 (0)	1.000
Hemodynamics on admission			
SBP, mm Hg	139.2 ± 38.4	145.4 ± 36.9	0.076
DBP, mm Hg	89.4 ± 21.5	95.4 ± 19.7	0.102
Heart rate	88.1 ± 17.3	93 ± 18.0	0.213
In-hospital complications			
Pulmonary infection	2 (4.0)	1 (8.3)	0.482
MI	1 (2.0)	0 (0)	1.000
Stroke	1 (2.0)	0 (0)	1.000
Cardiac tamponade	1 (2.0)	0 (0)	1.000
Aortic rupture	3 (6.0)	0 (0)	1.000
In-hospital mortality	5 (10.0)	0 (0)	0.573
One-year mortality	6 (12.0)	1 (8.3)	1.000

DBP, diastolic blood pressure; MM, medical management; SBP, systolic blood pressure; TEVAR, thoracic endovascular aortic repair.

Categorical variables are presented as number (%). Continuous variables are presented as mean ± standard deviation.

### Comparison of CT imaging between TEVAR group and MM group intype B IMH

To further investigate the differences in CT imaging characteristics between the two groups of patients with type B IMH, the following data were compared in this study: the number and location of intima lesions, the initial location and range of hematoma, the size of aorta and hematoma, CT value of hematoma, pleural effusion and other indicators and their reexamination results. The results showed that most of the patients in the TEVAR group had intima lesions (including ULP and PAU), which were significantly higher than those in the MM group (83.3% vs. 30.0%; *P* = 0.002). The intima lesions in the TEVAR group were mostly multiple, whereas the intima lesions in the MM group were mostly solitary. There was no significant difference between the two groups in the location of intimal lesions, which were mainly in the descending aorta. The proximal hematoma location was similar between the two groups, and most of them were located in the descending aorta (96.0% vs. 83.3%; *P* = 0.166). However, the TEVAR group had a wider range of hematoma involvement, with a higher proportion involving the iliac artery, and there was a significant difference between the two groups (33.3% vs. 2.0%, *P* = 0.004). The mean maximum diameter of the aorta in the TEVAR group was significantly larger than that in the MM group (43.6 mm ± 8.1 vs. 38.5 mm ± 7.2; *P* = 0.035). The maximum thickness of the hematoma was 14.9 ± 3.4 mm in the TEVAR group and 10.2 ± 2.8 mm in the MM group, the former was significantly larger than the latter (*P* = 0.043). There was no significant difference in the CT value of hematoma between the two groups, while the proportion of pleural effusion in the TEVAR group was significantly higher than that in the MM group (75.0% vs. 12.0%; *P* < 0.001). CT reexamination during hospitalization showed that a total of seven patients in the MM group had disease progression, including two patients with enlarged or thickened hematoma, and four patients with AD or aortic aneurysm. These seven patients were ultimately treated conservatively. The TEVAR group decided to receive TEVAR due to the progression of the lesion. The specific reasons were as follows: expanded hematoma scope and increased thickness in four patients, progression to AD in six patients, and increased pleural effusion in two patients. All these outcomes are depicted in Table 4.

**Table 4:** Ct imaging of TEVAR group and MM group.

Category	MM	TEVAR	*P* value
Number of patients	50	12	
Intima lesion (ULP/PAU)	15 (30.0)	10 (83.3)	0.002
Single lesion	12 (24.0)	2 (16.7)	0.872
Multiple lesions	3 (6.0)	8 (66.7)	<0.001
Location of major intima lesions			
Descending aorta	12 (24.0)	7 (58.3)	0.049
Abdominal aorta	3 (6.0)	3 (25.0)	0.146
Location of proximal hematoma			
Descending aorta	48 (96.0)	10 (83.3)	0.166
Abdominal aorta	2 (4.0)	2 (16.7)	0.166
Location of distal hematoma			
Descending aorta	13 (26.0)	3 (25.0)	1.000
Abdominal aorta	36 (72.0)	5 (41.7)	0.098
Iliac artery	1 (2.0)	4 (33.3)	0.004
MAD, mm	38.5 ± 7.2	43.6 ± 8.1	0.035
Maximum haematoma thickness, mm	10.2 ± 2.8	14.9 ± 3.4	0.043
CT value of hematoma, HU	65.3 ± 2.6	67.2 ± 4.6	0.156
Pleural effusion	6 (12.0)	9 (75.0)	<0.001
Reexamination of CT			
Disease progression	7 (14.0)	12 (100.0)	<0.001
Enlarged or thickened hematoma	2 (4.0)	4 (33.3)	0.011
Progression to AD or aneurysm	4 (8.0)	6 (50.0)	0.002
Increased pleural effusion	0 (0)	2 (16.7)	0.035
Other	1 (2.0)	0 (0)	1.000
No change or shrinkage of the hematoma	43 (86.0)	0 (0)	<0.001

AD, aortic dissection; MM, medical management; PAU, penetrating aortic ulcer; TEVAR, thoracic endovascular aortic repair; ULP, ulcer-like projection.

Categorical variables are presented as number (%). Continuous variables are presented as mean ± standard deviation.

## Discussion

IMH is one of the common emergencies in vascular surgery. According to the International Registration of Aortic Dissection (IRAD) statistics, about 58% of IMH patients are classified as Stanford type B (8). This study first compared the clinical characteristics of type B IMH and type B AD. There were significant differences in gender composition between the two groups, and the proportion of males in AD group was significantly higher. The average age of the IMH group was significantly higher than that of the AD group, suggesting that the age of onset of IMH was later or the progression of the disease was slower. Studies have shown that female patients with acute aortic syndrome are older, with atypical symptoms and high mortality (9). The comparison of risk factors and common comorbidities between the two groups showed that there was no significant difference between the two groups except for chronic renal insufficiency. This may be due to the fact that the lesions of some AD patients involved the renal artery, while IMH was less likely to have poor organ perfusion and had a better long-term prognosis (10). The initial symptoms were mainly chest and back pain, and other symptoms included abdominal pain, loss of consciousness, dyspnea and so on. The admission CT images of the patients showed that AD lesions involved a wider range, and the proportion of involved iliac arteries was significantly higher than that in the IMH group. Considering the pathogenesis of AD, high-pressure blood flow travels under the intima of the rupture, so it may involve a wider range. While the hematoma of IMH is limited by the adventitia and branch vessels, the range of involvement is smaller.

The current mainstream view for type B IMH is that conservative treatment is the mainstay of the disease (11). In fact, some patients will eventually develop into dissection, aneurysm or even rupture during the follow-up period (12). The clinical and CT imaging characteristics that suggest the need for TEVAR are the focus of this study. Among the 62 IMH patients, 12 patients were finally treated with TEVAR. There was no significant difference in gender composition and age between TEVAR group and MM group. The proportion of patients with dyslipidemia in the TEVAR group was significantly increased, and increased blood lipids is one of the risk factors for endometrial lesions (such as atherosclerosis). Studies have found that if IMH has the following high-risk signs on CT imaging: ULP, increased diameter of aorta, increased thickness of hematoma, intermural blood pool and pericardium/pleural effusion, it is easy to progress to AD, aneurysm or rupture (4). IMH has a unique pathophysiological mechanism compared with AD. In addition to the traditional viewpoint that IMH originates from the rupture of the vasa vasorum, a series of studies have confirmed that a considerable part of IMH is AD with thrombosis of the false lumen. Microscopic intimal rupture can be seen in CT images, which can be further confirmed by reexamination of CTA. The CT value of a typical IMH hematoma is usually 60–70HU, and the thickness of the hematoma is generally greater than 7 mm (13). In this study, most patients in the TEVAR group had intima lesions (including ULP and PAU), and most of them were multiple lesions, which increased significantly compared with the MM group and were mainly located in the descending aorta. The TEVAR group involved a wider range of hematoma and a higher proportion involved the iliac artery. The maximum diameter of aorta, the thickness of hematoma and the ratio of pleural effusion in the TEVAR group were significantly higher than those in the MM group. The above indicators are all risk factors suggesting that the progression of IMH requires surgical intervention, and should be paid close attention in future clinical work. A number of studies have identified high-risk CTA signs of IMH progression (14). Schlatter et al. believed that ULP was associated with complications of IMH, including aneurysm, dissection, rupture, vessel wall thickening, increased surgery rate and mortality. The diameter of ULP is in the range of 10–20 mm and the depth is in the range of 5–10 mm, which is related to the progression of dissection, aneurysm and rupture. Hematoma thickness greater than 11–16 mm is associated with complications (15). Park et al.'s study suggested that type B IMH with a diameter greater than 41 mm is associated with a higher risk (16). Wu et al.'s study suggested that pleural/pericardial effusion was related to IMH complications, such as dissection, aneurysm, surgery and death (17).

All IMH patients were initially treated with medication on admission, primarily to control blood pressure, heart rate and pain. As previously mentioned, a significant proportion of IMH is pseudoluminal thrombotic AD, and many cases are associated with intimal lesions such as ULP and PAU. In the case of unstable blood pressure control, the shear force of the intima impacted by blood flow becomes larger, and the original intima lesions may form a rupture or the previously closed rupture may reopen, forming AD. This further suggests the importance of blood pressure control in IMH medical management (10).

IRAD data show that 5% of Stanford type B IMH require surgical treatment. The main indications are pain, uncontrollable blood pressure, increased thickness of hematoma, combined with aortic PAU, false lumen oppressing the true lumen or various sign of rupture. Active endovascular stent treatment for such Stanford type B IMH was recommended (18). According to the latest 2022 ACC/AHA guidelines, in patients with uncomplicated type B IMH, medical therapy as the initial management strategy is recommended (19). Erbel et al.'s study showed that 61%–91% of patients with uncomplicated IMH had stable or reduced hematoma after medical management, and when complicated conditions such as pericardial effusion, shock, and aneurysm occurred, surgical intervention should be actively considered (20). In this study, all patients in the TEVAR group were treated with TEVAR due to disease progression, the specific reasons included the enlarged or thickened hematoma in four patients, the progression to AD in six patients, and the increase of pleural effusion in two patients. The surgical indications were similar to those in the above literature, and no death occurred in patients treated with TEVAR. A total of seven patients in the MM group had disease progression, including two with hematoma expansion and thickness increase, four with AD or aortic aneurysm. After clinical and imaging evaluation, they were found to be unsuitable for TEVAR. Five patients died in the MM group (three from aortic rupture, one from cerebral infarction, and one from esophageal cancer). The above data suggest that for patients with Stanford type B IMH, it is not only necessary to detect high-risk factors in CT imaging for surgical intervention in time, for patients who do not have these risk factors temporarily, follow-up should also be strengthened.

A systematic analysis showed that the in-hospital mortality of type B AD with different treatment methods was 0%–27% (median 7%) with medical management, 13%–17% (median 16%) with open surgery, and 0%–18% (median 6%) with TEVAR (8). Mesar et al. treated 67 type B IMH patients with medical management after admission, 34 patients failed within 14 days of admission, and 14 patients failed after 14 days of admission. Finally, only 19 patients were successfully treated, and the failure rate of medical management was 71.6%. While the thickness of intramural hematoma was an important factor for medical management failure (21). In the studies of Bischoff et al. and Schoenhoff et al., the failure rate of medical management in patients with type B IMH was 68.3% and 60.0%, respectively. These studies showed that the failure rate of medical management alone was higher (22, 23).

In a multicenter retrospective study of 41 patients with type B IMH, 31 patients were treated with TEVAR and 10 patients were treated with medical management alone. During the 12-month follow-up period, survival rate was lower in the medical management group than in the TEVAR group (77% vs. 85%), with no statistical difference between the two groups. However, patients in the TEVAR group had significantly lower rates of aortic disease progression and adverse aortic events than in the medical management group (24). Li et al. included 56 type B IMH patients in their study, and considered that TEVAR was suitable for patients with the maximum diameter of aorta above 45 mm, the thickness of hematoma above 10 mm, and patients with persistent chest and back pain after drug treatment. All 33 patients in the TEVAR group were successfully operated, and there was no death in hospital. Of the 23 patients in the medical management group, six patients progressed to AD and two died (25). A meta-analysis comparing TEVAR and medical management, including nine studies with a total of 327 patients with type B IMH, showed that TEVAR treatment reduced the probability of long-term progression to dissection and aortic rupture (26). It is worth noting that there are also some unfavorable factors in the treatment of IMH with TEVAR, mainly due to the weak blood vessel wall caused by the hematoma, which may not be able to withstand the pressure of the balloon expansion and stent release during the TEVAR surgery. TEVAR surgery also has a series of other risks, so it is not advisable to blindly expand the indications (27).

## Conclusion

In conclusion, Stanford type B IMH is one of the common emergencies in vascular surgery, and it has certain pathophysiological characteristics compared with Stanford type B AD. In the diagnosis of IMH, patients' symptoms and high-risk signs of CTA should be paid attention to. TEVAR therapy should be actively considered on the basis of effective medical management when there are intima lesions (ULP/PAU), increased aortic diameter and hematoma thickness, extensive hematoma involvement, and pleural effusion.

## Data Availability

The original contributions presented in the study are included in the article/Supplementary Material, further inquiries can be directed to the corresponding author/s.
